# Preparation of P-C_3_N_4_/red mud composites for enhanced photo-Fenton degradation of rhodamine B

**DOI:** 10.1039/d6ra08398f

**Published:** 2026-10-22

**Authors:** Chao Wu, Ying Wu

**Affiliations:** a School of Chemistry and Chemical Engineering, Tarim University Aral Xinjiang 843300 China wuyingjuyuan@163.com; b South Xinjiang Chemical Resource Utilization Engineering Laboratory, Xinjiang Production and Construction Corps Aral Xinjiang 843300 China

## Abstract

Red mud (RM), a bauxite residue with high alkalinity and abundant iron phases, remains underutilized. Herein, a heterogeneous photo-Fenton catalyst was constructed by integrating phosphorus-doped graphitic carbon nitride (P-C_3_N_4_) with RM *via* an impregnation-thermal route. Characterizations confirmed P incorporation into the heptazine network and robust interfacial interaction between P-C_3_N_4_ and iron-rich RM components (hematite and goethite). The optimized P-C_3_N_4_/RM (3) composite exhibited an apparent band gap of 1.6 eV, markedly enhanced visible-light absorption, and improved photocurrent response *versus* pristine g-C_3_N_4_ (2.7 eV). Under visible-light/H_2_O_2_ conditions, the catalyst achieved 99.7% rhodamine B degradation within 120 min of irradiation, with an apparent rate constant of 0.036 min^−1^, substantially outperforming bare g-C_3_N_4_, P-C_3_N_4_, and pristine RM. The system maintained high efficiency in the pH 3–6 range and remained active in the pH 7–9 range, and it also showed good tolerance to coexisting anions and humic acid. After three consecutive cycles, the catalyst retained 96% activity. Radical quenching and ESR analyses identified ·OH and ^1^O_2_ as the dominant reactive species, with Fe^2+^/Fe^3+^ cycling sustaining H_2_O_2_ activation. This work offers a waste-derived catalytic platform that simultaneously valorizes RM and treats dye-laden wastewater, providing a sustainable strategy for industrial solid waste management.

## Abstract 2

A waste-derived P-C_3_N_4_/RM photo-Fenton catalyst was developed. The synergy between P doping and red mud enables efficient RhB degradation *via* Fe^3+^/Fe^2+^ cycling and ·OH/^1^O_2_.

## Graphical abstract

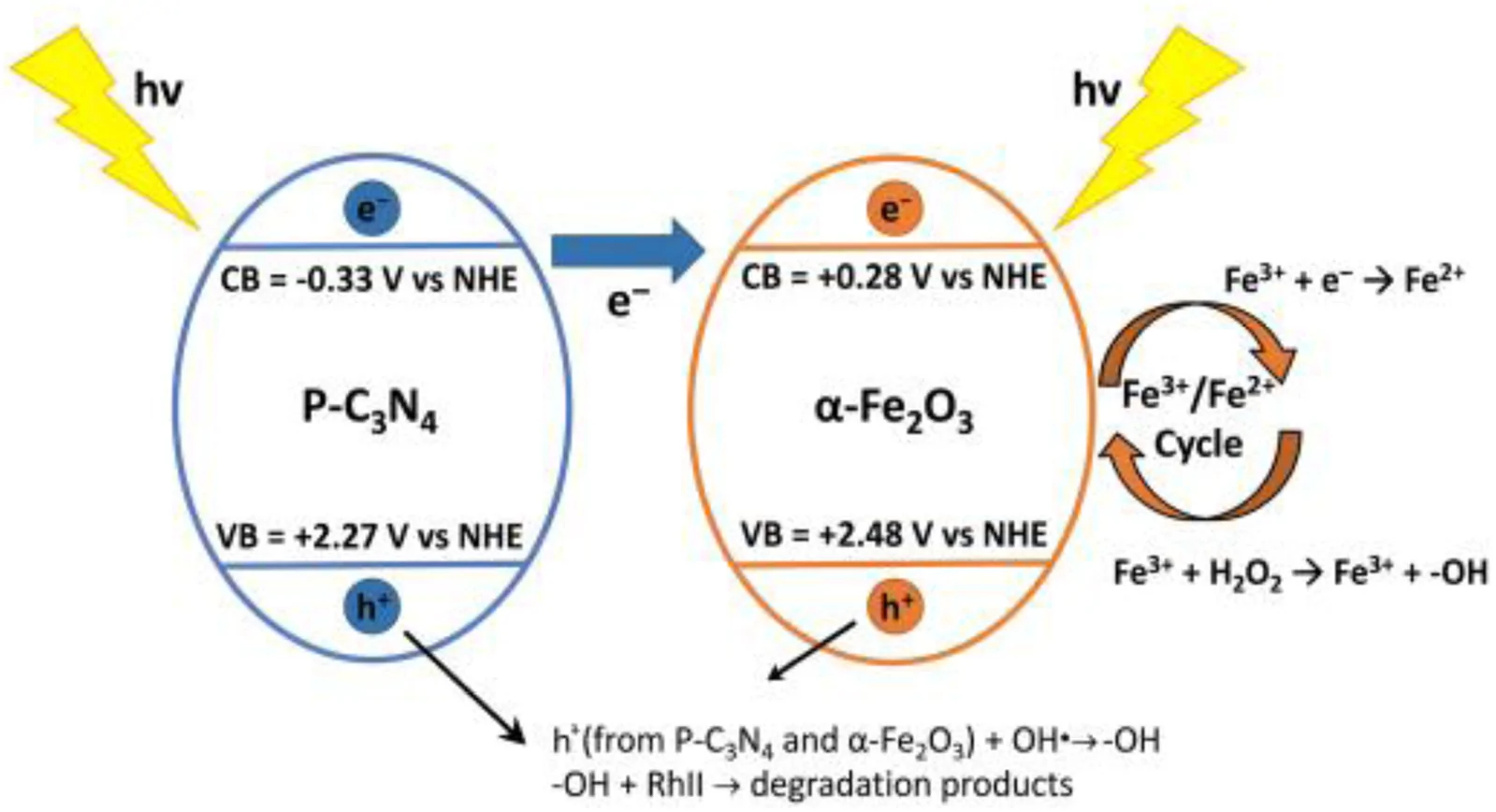

## 1. Introduction

Advanced oxidation processes (AOPs) are widely regarded as effective technologies for the treatment of refractory organic wastewater because they generate highly oxidative reactive oxygen species capable of mineralizing persistent contaminants.^1^ Among these technologies, Fenton oxidation has attracted considerable attention owing to its operational simplicity and strong oxidation capability. However, conventional homogeneous Fenton processes suffer from a narrow effective pH window (typically pH 2–4), substantial iron-sludge generation, and inefficient Fe^3+^/Fe^2+^ cycling, which restrict their practical application.^2^

Conventional Fenton treatment generally requires the addition of soluble ferrous salts. In contrast, red mud (RM), a strongly alkaline solid residue from alumina production, is generated in enormous quantities and poses persistent environmental risks. Its high contents of Fe_2_O_3_ (typically 20–50%) and Al_2_O_3_, however, make it a potentially inexpensive iron source for heterogeneous Fenton catalysis. Converting RM into a catalytic component can therefore combine solid-waste valorization with a reduction in catalyst cost.^3–5^

Graphitic carbon nitride (g-C_3_N_4_) is a metal-free polymeric semiconductor with an appropriate band gap (∼2.7 eV), visible-light response, good chemical stability, and low-cost precursors, and has consequently been extensively investigated for photocatalysis. Nevertheless, pristine g-C_3_N_4_ is limited by rapid recombination of photogenerated charge carriers and a relatively low specific surface area, making rational modification necessary to improve its catalytic performance.

## 2. Materials and methods

### 2.1. Materials and instrumentation

Red mud (RM) was supplied by Shandong Yizhu Materials Co., Ltd. Urea (CH_4_N_2_O, analytical grade), hydrochloric acid (HCl, analytical grade), and hydrogen peroxide (H_2_O_2_, analytical grade) were purchased from Sinopharm Chemical Reagent Co., Ltd. Hexachlorocyclotriphosphazene (HCCP, analytical grade) was obtained from Shanghai Macklin Biochemical Co., Ltd, and anhydrous ethanol (analytical grade) was purchased from Tianjin Kermel Chemical Reagent Co., Ltd.

Electrochemical measurements were carried out using a CHI760E electrochemical workstation (Shanghai Chenhua Instrument Co., Ltd). Particle-size/zeta-potential measurements were performed using a Zetasizer Advance ZEN3700 (Malvern Panalytical). X-ray diffraction (XRD) patterns were recorded on a SmartLab SE diffractometer (Rigaku, Japan). UV-vis spectra were obtained using a UV-1900i spectrophotometer. Fourier-transform infrared (FTIR) spectra were collected with an IRSpirit spectrometer (Shimadzu). X-ray photoelectron spectroscopy (XPS) was performed using a PHI GENESiS 500 instrument, and transmission electron microscopy (TEM) was conducted on a JEM-2100Plus microscope (JEOL, Japan).

### 2.2. Catalyst preparation

#### 2.2.1. Synthesis of P-C_3_N_4_

Urea (3 g) and HCCP (0.075 g) were ground and mixed thoroughly, transferred to a three-neck flask, and dispersed in deionized water (5 mL), anhydrous ethanol (5 mL), and HCl (0.1 M, 1 mL). After stirring for 1 h at room temperature, the mixture was heated to 100 °C until a viscous precursor was obtained. The precursor was transferred to a crucible and dried at 70 °C for 12 h. The dried sample was then heated in a muffle furnace to 500 °C at a programmed rate of 100 °C per 30 min and held at 500 °C for 3 h. After cooling to room temperature, the product was weighed and ground to obtain phosphorus-doped carbon nitride (P-C_3_N_4_).

#### 2.2.2. Synthesis of P-C_3_N_4_/RM composites

Urea (3 g), HCCP (0.075 g), and different amounts of RM (0.2, 0.5, 0.8, and 1.0 g) were thoroughly ground and mixed, transferred to a three-neck flask, and dispersed in deionized water (5 mL), anhydrous ethanol (5 mL), and HCl (0.1 M, 1 mL). After stirring for 1 h at room temperature, the suspension was heated to 100 °C until it became viscous and was then transferred to a crucible for drying at 70 °C for 12 h. The dried precursor was heated to 500 °C in a muffle furnace at a programmed rate of 100 °C per 30 min and held at 500 °C for 3 h. After cooling, the products were weighed and ground. The resulting composites were denoted P-C_3_N_4_/RM (1), P-C_3_N_4_/RM (2), P-C_3_N_4_/RM (3), and P-C_3_N_4_/RM (4), respectively (Fig. 1).

**Fig. 1:**
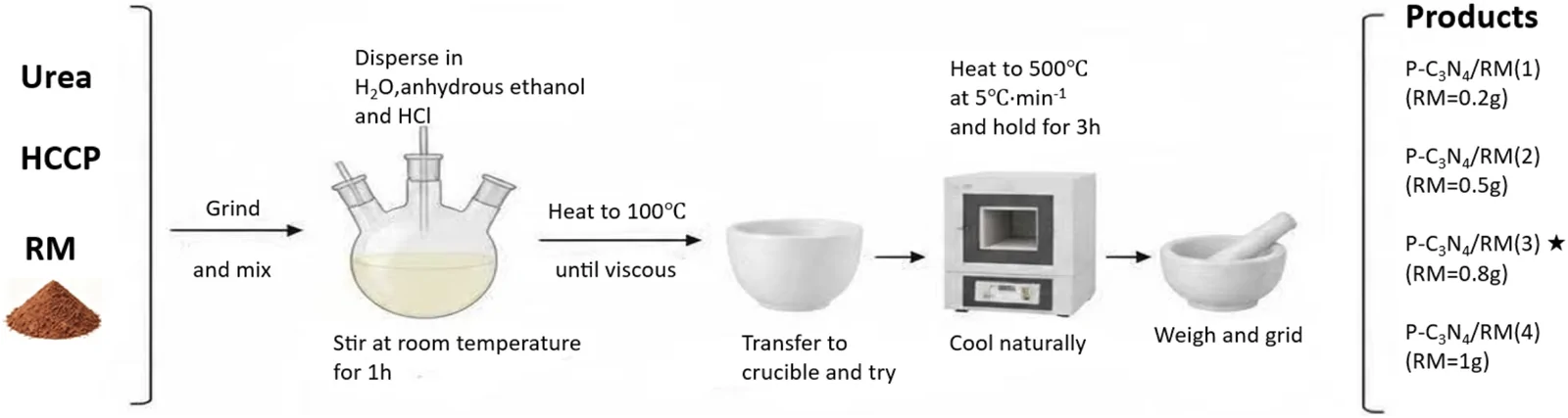
Schematic illustration of the synthesis route for P-C_3_N_4_/RM (1–4) composites.

### 2.3. Characterization

The phase structure, morphology, elemental composition, surface chemical states, and optical properties of the materials were characterized by XRD, scanning electron microscopy (SEM), energy-dispersive X-ray spectroscopy (EDS), FTIR spectroscopy, UV-vis diffuse-reflectance spectroscopy (UV-vis DRS), photoluminescence (PL) spectroscopy, XPS, and electron microscopy, as appropriate.

### 2.4. Photo-Fenton degradation experiments

Rhodamine B (RhB) was used as the model organic pollutant. The RhB concentration was 1 × 10^5^ M and the solution volume was 60 mL. The initial pH was adjusted using HCl or NaOH, and the effect of pH was investigated over the range of 3–9. Before catalyst screening, three systems, *i.e.*, Fenton, photocatalysis, and photo-Fenton, were compared under the same conditions. The photo-Fenton system showed the highest degradation efficiency and was therefore selected for further catalyst screening. For catalyst screening, 20 mg of each catalyst was first used to identify the optimal composite, and P-C_3_N_4_/RM (3) was selected. The catalyst dosage was then further optimized using 5, 10, 15, and 20 mg of P-C_3_N_4_/RM (3), corresponding to 0.083, 0.167, 0.25, and 0.333 g L^−1^, respectively. The suspension was stirred in the dark for 30 min to establish adsorption–desorption equilibrium. Subsequently, 5 µL of 30 wt% H_2_O_2_ was added, and the suspension was immediately irradiated with a 300 W Xe lamp at a focused light intensity of approximately 89 mW cm^−2^ under continuous stirring, without a cutoff filter. The reaction was carried out at room temperature. Aliquots were withdrawn every 30 min over a total irradiation time of 120 min, and the RhB concentration was determined from the absorbance at 554 nm, the maximum absorption wavelength of RhB. Based on the Beer–Lambert relationship, the degradation efficiency, *D*, was calculated using eqn (1):1
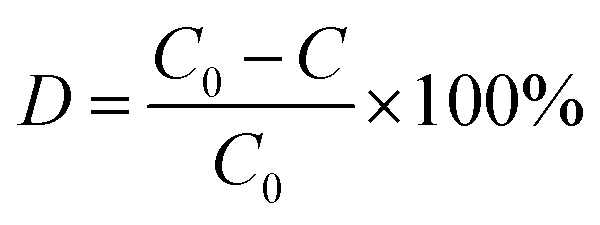
where *D* is the RhB degradation efficiency (%), and *C*_0_ and *C* are the RhB concentrations (or proportional absorbance values) before reaction and at reaction time *t*, respectively.

The apparent pseudo-first-order rate constant was determined using eqn (2):2
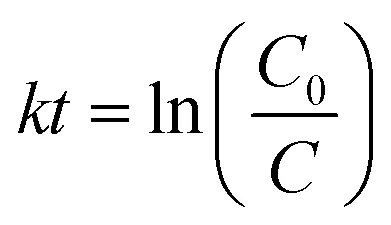
where *k* is the apparent reaction rate constant (min^−1^) and *t* is the reaction time (min).

### 2.5. Photoelectrochemical measurements

Photoelectrochemical measurements were performed in a conventional three-electrode cell using Pt as the counter electrode, saturated Ag/AgCl as the reference electrode, and fluorine-doped tin oxide (FTO) glass as the working electrode. The catalyst and 5 wt% Nafion were dispersed in ethanol by ultrasonication to form a homogeneous slurry, which was coated onto the FTO substrate and dried at 150 °C for 60 min. A 0.5 mol L^−1^ Na_2_SO_4_ solution (pH 6.8) was used as the supporting electrolyte. A 300 W Xe lamp served as the visible-light source. Transient photocurrent responses were recorded at 0.5 V *versus* Ag/AgCl. Electrochemical impedance spectra (EIS) were collected at −0.3 V *versus* Ag/AgCl over a frequency range of 10 kHz to 0.01 Hz using a 10 mV AC perturbation.

## 3. Results and discussion

### 3.1. Chemical composition of red mud

The bulk chemical composition of the raw RM was determined by X-ray fluorescence (XRF), and the results are summarized in Table 1. The material consists mainly of metal and non-metal oxides, with Fe_2_O_3_, Al_2_O_3_, SiO_2_, and Na_2_O as the dominant components. In particular, the high Fe_2_O_3_ content provides a substantial intrinsic iron reservoir for heterogeneous Fenton reactions.

**Table 1:** Main chemical composition of the red mud

No.	Component	Content	Unit
1	Fe_2_O_3_	44.7	Mass%
2	Al_2_O_3_	22.6	Mass%
3	SiO_2_	14.6	Mass%
4	Na_2_O	10.1	Mass%
5	CaO	0.867	Mass%

### 3.2. Structural and morphological characterization of P-C_3_N_4_/RM

SEM images at different magnifications (Fig. 2a and b) show the typical lamellar and porous morphology of carbon nitride, with RM particles distributed on or partially embedded in the C_3_N_4_ matrix, forming close interfacial contact. EDS analysis (Fig. 2c) confirms the simultaneous presence of C, P, Fe, O, Si, and Al in the composite. The XRD patterns of g-C_3_N_4_, P-C_3_N_4_, and P-C_3_N_4_/RM are compared in Fig. 2d. Pristine g-C_3_N_4_ exhibits characteristic diffraction peaks at 2*θ* ≈ 13° and 27.5°, assigned to the (100) in-plane structural packing and the (002) interlayer stacking of the graphitic structure, respectively. After P doping, these characteristic peaks are retained, while the (002) peak becomes broader and weaker, indicating that incorporation of P perturbs the periodic stacking of the carbon nitride layers.^6^ After RM loading, additional reflections associated with iron-containing crystalline phases in RM, including Fe_2_O_3_, appear together with the C_3_N_4_ reflections, confirming the successful formation of the composite without destroying the principal Fe_2_O_3_ crystalline phase.

**Fig. 2:**
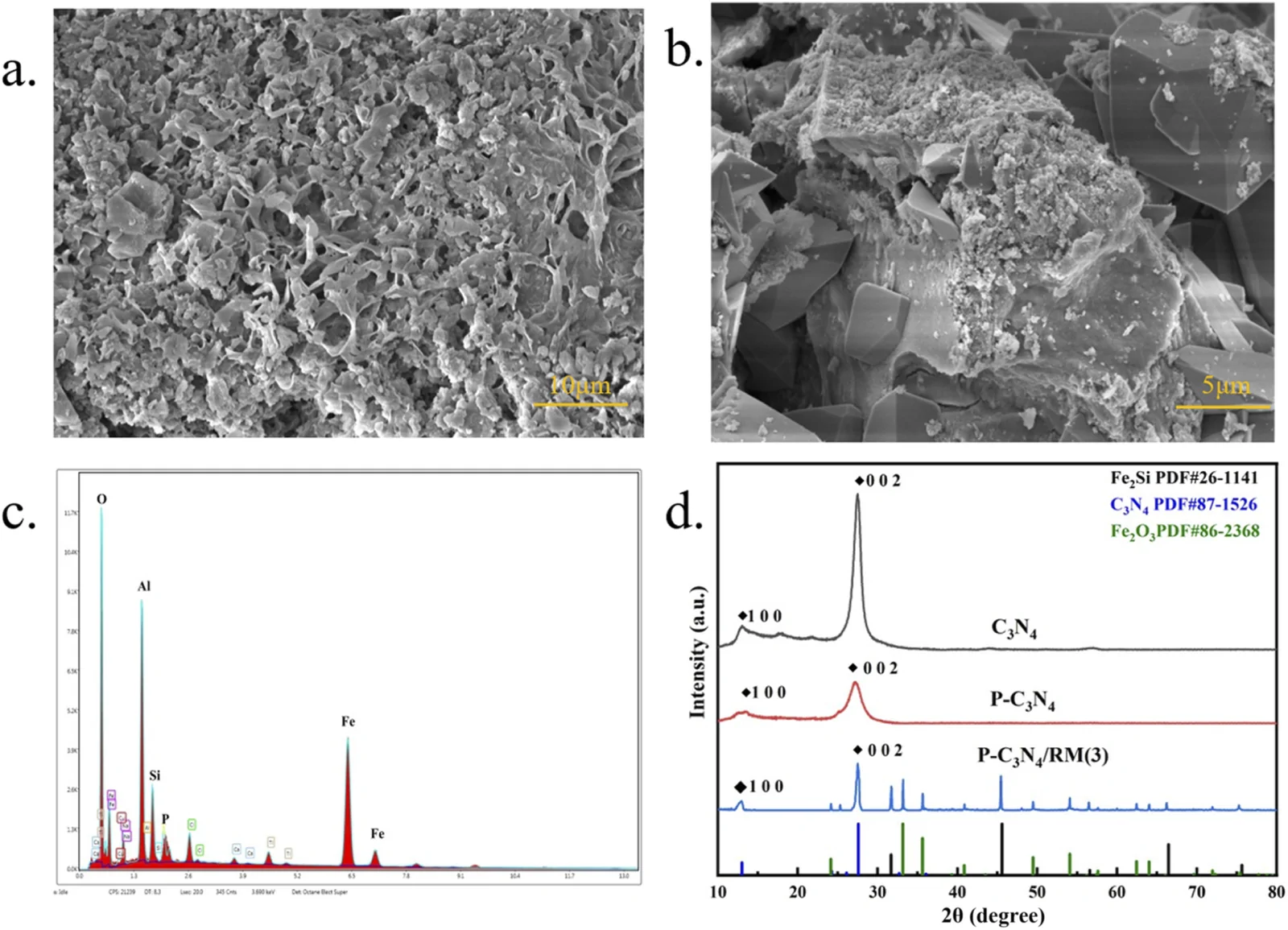
Morphology, elemental analysis, and XRD patterns of P-C_3_N_4_/RM: (a and b) SEM images at different magnifications; (c) EDS spectrum; and (d) XRD patterns.

The synthesized materials were characterized by Fourier transform infrared spectroscopy (FTIR). As shown in Fig. 3, P-C_3_N_4_ exhibits the characteristic absorption peaks of g-C_3_N_4_ at 805.7 cm^−1^, 1349.4 cm^−1^, and 3547.2 cm^−1^. The broad band in the range of 3100–3400 cm^−1^ is attributed to the N–H stretching vibration of free amino groups, while the absorption bands between 1250 and 1634 cm^−1^ correspond to the typical stretching vibrations of the C–N heterocyclic ring. The peak at 810 cm^−1^ is assigned to the stretching vibration of the tri-*s*-triazine ring. The presence of these characteristic peaks unequivocally confirms the integrity of the g-C_3_N_4_ framework structure in P-C_3_N_4_. Compared with pristine g-C_3_N_4_, no significant shift in the characteristic peak positions of P-C_3_N_4_ is observed, indicating that phosphorus doping does not disrupt the fundamental crystal structure. Nevertheless, subtle variations in the intensity of certain peaks imply a redistribution of electron cloud density, which may be associated with the incorporation of phosphorus atoms. In addition to the aforementioned characteristic peaks, two new absorption bands appear at 532 cm^−1^ and 465.2 cm^−1^. The absorption peak at 446.8 cm^−1^ corresponds to the Fe–O bond in red mud (RM), while the band at 465.2 cm^−1^ originates from the vibration of Si–O–Si bridging bonds. These results demonstrate that red mud has been successfully loaded onto the phosphorus-doped carbon nitride (P-C_3_N_4_) substrate, confirming the successful construction of the P-C_3_N_4_/RM (3) composite catalyst.

**Fig. 3:**
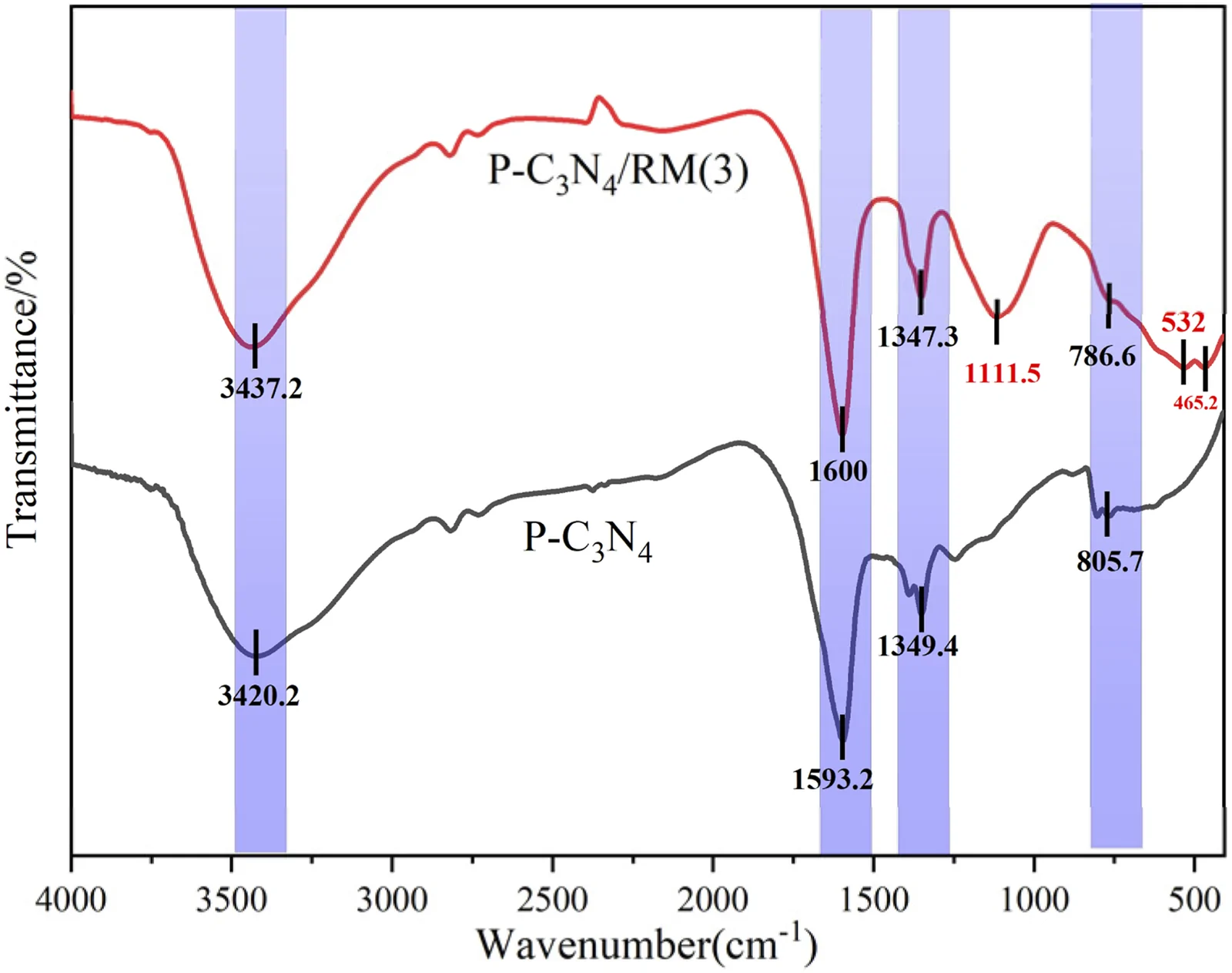
FTIR spectra of P-C_3_N_4_ and P-C_3_N_4_/RM (3).

XPS was employed to investigate the elemental composition and chemical states of the composite. The survey spectrum (Fig. 4a) clearly shows O, Fe, C, Al, and P signals, confirming the incorporation of both RM-derived elements and phosphorus into the carbon nitride-based material. In the high-resolution Fe 2p spectrum (Fig. 4b), the main peaks located at approximately 710.5 and 724.2 eV are assigned to Fe 2p_3/2_ and Fe 2p_1/2_, respectively. The peak shape and satellite features indicate the coexistence of Fe^2+^ and Fe^3+^, consistent with iron-oxide phases in RM and favorable for Fe^3+^/Fe^2+^ redox cycling. The O 1s spectrum (Fig. 4c) can be deconvoluted into contributions from lattice oxygen (∼530.2 eV), adsorbed/surface oxygen (∼531.5 eV), and carbonyl-related oxygen (∼533.1 eV), reflecting a chemically heterogeneous oxygen environment at the interface. The Al 2p signal at ∼74.3 eV (Fig. 4d) is consistent with Al_2_O_3_-type species. Importantly, the P 2p spectrum (Fig. 4e) contains a doublet in the 133.5–135 eV region. The reported binding energy near 133.8 eV is consistent with P–N bonding, whereas P–C bonds are typically observed at lower binding energies (approximately 131.5–132.5 eV).^7^ These results support the successful incorporation of phosphorus into the g-C_3_N_4_ framework and the establishment of interfacial chemical interactions in P-C_3_N_4_/RM.

**Fig. 4:**
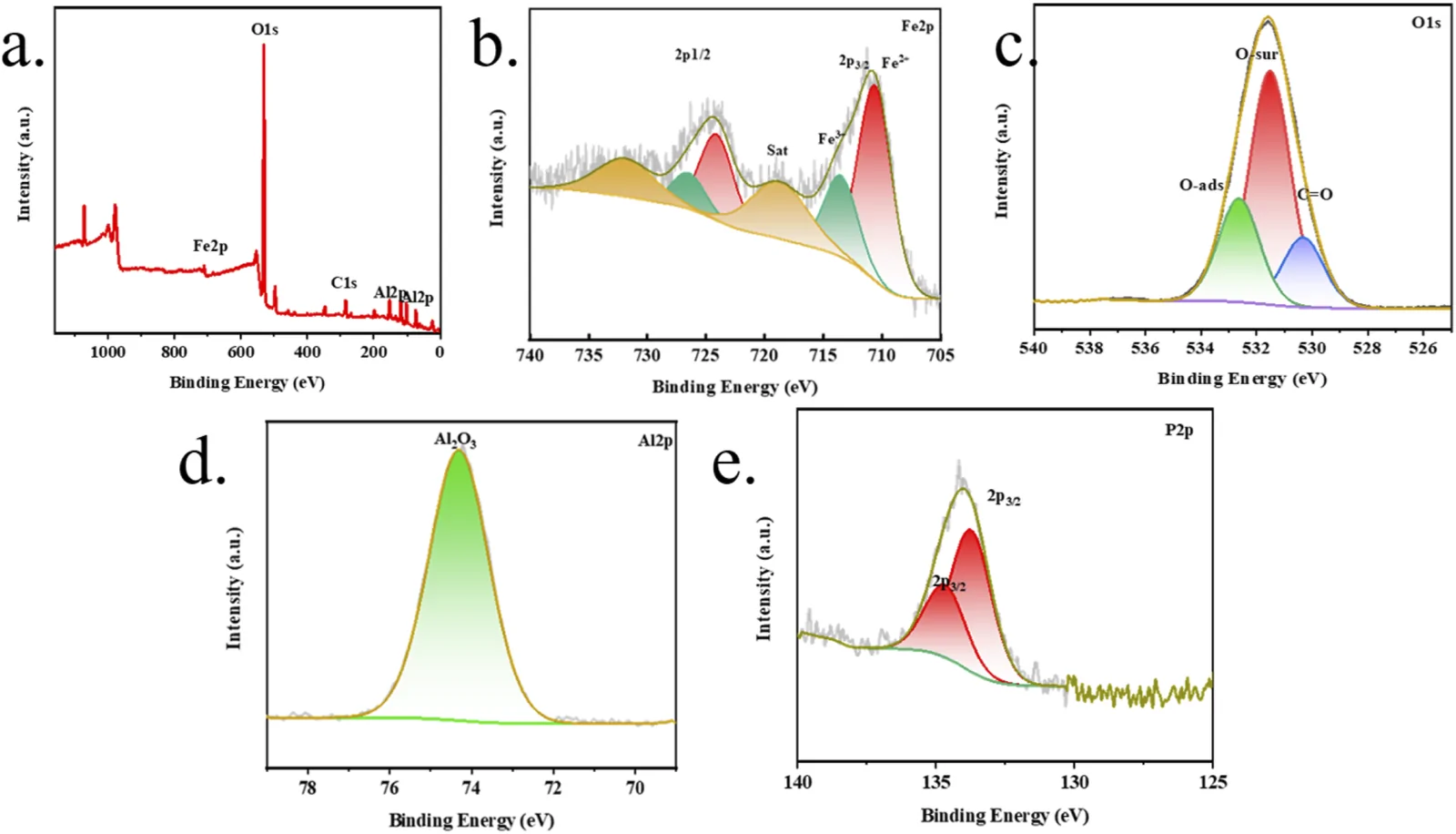
XPS spectra of P-C_3_N_4_/RM: (a) survey spectrum; (b) Fe 2p; (c) O 1s; (d) Al 2p; and (e) P 2p.

The light-harvesting and charge-recombination behaviors of g-C_3_N_4_, P-C_3_N_4_, and P-C_3_N_4_/RM were evaluated by UV-vis DRS and PL spectroscopy (Fig. 5). Pristine g-C_3_N_4_ and P-C_3_N_4_ show absorption edges in the visible-light region, whereas P-C_3_N_4_/RM exhibits substantially extended and intensified visible-light absorption. The band gaps were estimated by Tauc-plot analysis using an indirect allowed transition model, in which (*αhv*)^1/2^ was plotted against *hv*, and the linear region near the absorption edge was extrapolated to the baseline. The apparent band gaps of g-C_3_N_4_, P-C_3_N_4_, and P-C_3_N_4_/RM are estimated to be 2.7, 2.6, and 1.6 eV, respectively. It should be noted that the apparent band gap of P-C_3_N_4_/RM cannot be attributed solely to P doping. Fe_2_O_3_ and other inorganic phases in RM strongly absorb visible light and contribute significantly to the apparent absorption edge of the composite. Therefore, the value of 1.6 eV should be regarded as the apparent optical band gap of P-C_3_N_4_/RM rather than the intrinsic band gap of P-C_3_N_4_ alone. In addition, P doping decreases the PL intensity relative to pristine g-C_3_N_4_, indicating suppressed electron–hole recombination. The PL emission of P-C_3_N_4_/RM is further quenched compared with P-C_3_N_4_, suggesting more efficient interfacial separation and transfer of photogenerated charge carriers after coupling with RM.

**Fig. 5:**
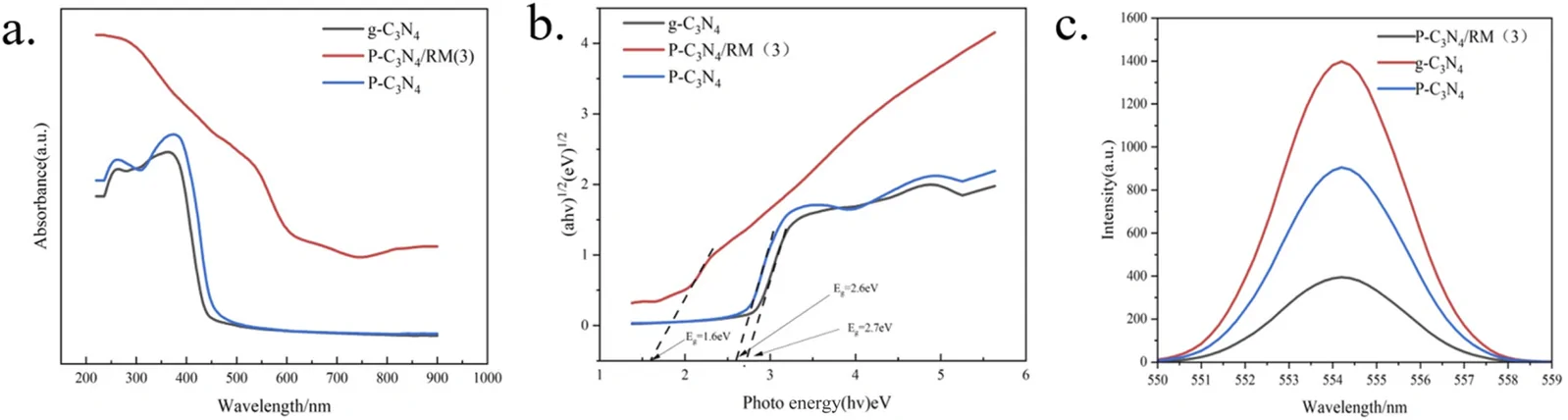
Optical properties of g-C_3_N_4_, P-C_3_N_4_, and P-C_3_N_4_/RM (3): (a) UV-vis diffuse-reflectance spectra, (b) Tauc plots, and (c) PL spectra.

### 3.3. Photoelectrochemical properties

Transient photocurrent and EIS measurements were used to probe charge separation and interfacial transport in g-C_3_N_4_, P-C_3_N_4_ and P-C_3_N_4_/RM (3) (Fig. 6a and b). All samples exhibit rapid and reproducible on/off photocurrent responses under intermittent irradiation. P-C_3_N_4_/RM (3) produces the highest photocurrent density, approximately twice that of pristine g-C_3_N_4_, indicating the most efficient separation of photogenerated carriers. Consistently, the Nyquist plot of P-C_3_N_4_/RM (3) shows the smallest semicircle radius, corresponding to the lowest interfacial charge-transfer resistance.

**Fig. 6:**
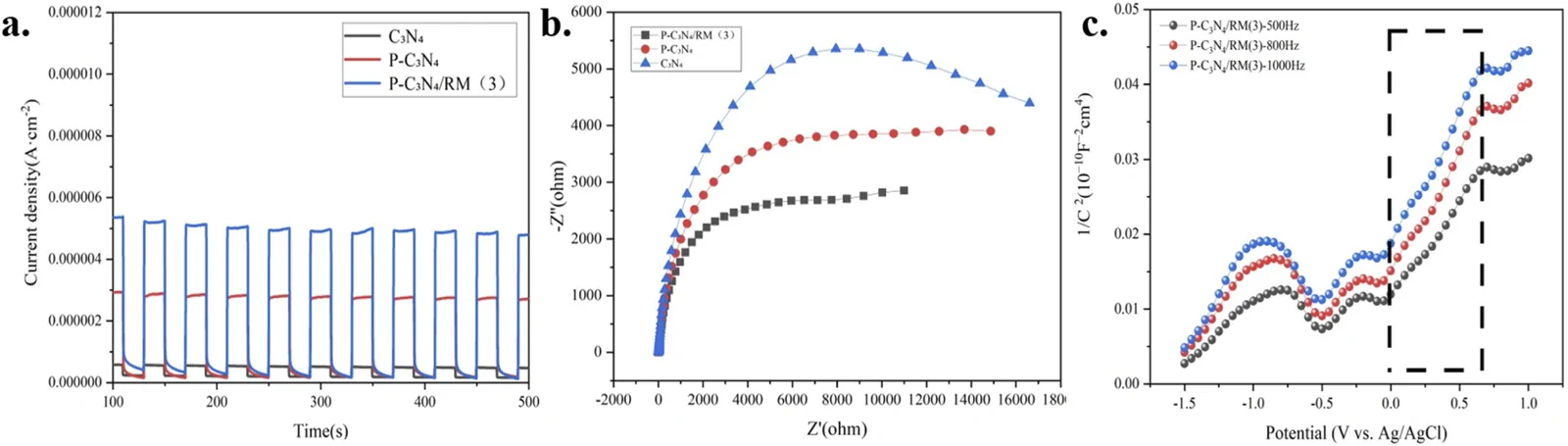
Photoelectrochemical characterization of P-C_3_N_4_/RM (3): (a) transient photocurrent responses of g-C_3_N_4_, P-C_3_N_4_ and P-C_3_N_4_/RM (3); (b) electrochemical impedance spectra; and (c) Mott–Schottky plots of P-C_3_N_4_/RM (3) at 500, 800 and 1000 Hz. The dashed box indicates the linear region used for flat-band potential extrapolation.

To further determine the band position of P-C_3_N_4_/RM (3), Mott–Schottky measurements were carried out at 500, 800 and 1000 Hz (Fig. 6c). The M–S plot exhibits three linear regions with different slopes, which is related to the multi-component semiconductor character of the composite. The linear region at 0.0–0.6 V *vs.* Ag/AgCl, marked by the dashed box, was selected for flat-band potential estimation because it shows the best linearity and the smallest frequency dependence, indicating that this region is dominated by the space-charge response rather than surface states. In addition, the 1000 Hz data were used because higher frequencies effectively suppress the contribution of surface states and interfacial states, giving a flat-band potential that better reflects the intrinsic space-charge behavior of the composite. The values obtained at 500, 800 and 1000 Hz were about −0.26, −0.25 and −0.33 V *vs.* NHE, respectively. The value at 1000 Hz (−0.33 V *vs.* NHE) was used to estimate the conduction-band position of P-C_3_N_4_/RM (3).

### 3.4. Photo-Fenton degradation performance

Before irradiation, all suspensions were stirred in the dark for 30 min to establish adsorption–desorption equilibrium. As shown in the dark segments of Fig. 7–11, the change in RhB concentration during this period was mainly attributed to adsorption on the catalyst surface rather than degradation, and the extent of dark adsorption depended on the catalyst and reaction system. After the dark period, no obvious further change was observed, confirming that adsorption–desorption equilibrium was reached. The initial concentration *C*_0_ used for calculating *C*/*C*_0_ was the RhB concentration before dark adsorption, and the concentration at the end of the dark period was taken as the starting point for the subsequent photo-Fenton degradation.

**Fig. 7:**
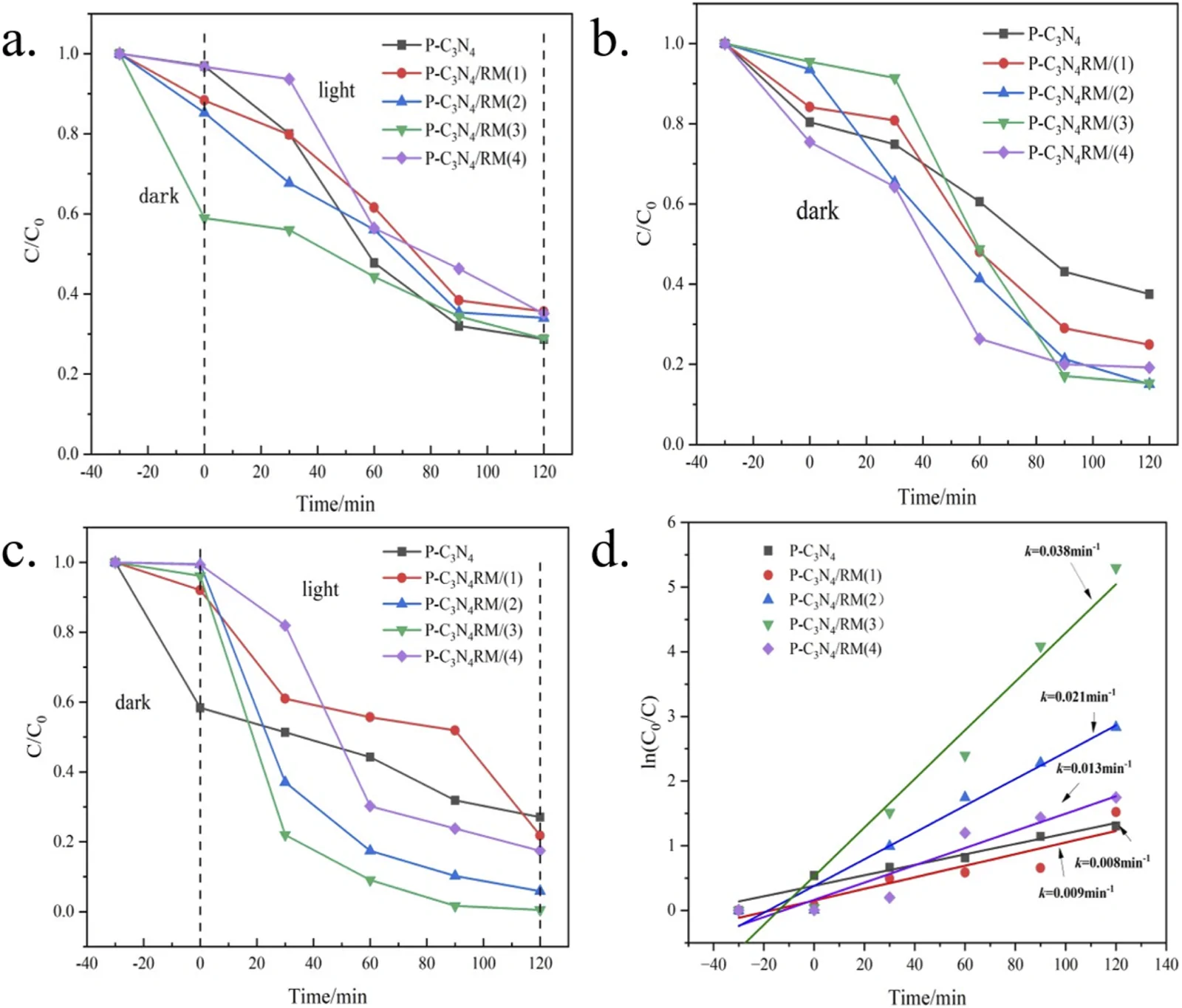
RhB degradation over g-C_3_N_4_, P-C_3_N_4_, and P-C_3_N_4_/RM under (a) photocatalytic, (b) Fenton, and (c) photo-Fenton conditions, and (d) corresponding pseudo-first-order kinetic plots.

**Fig. 8:**
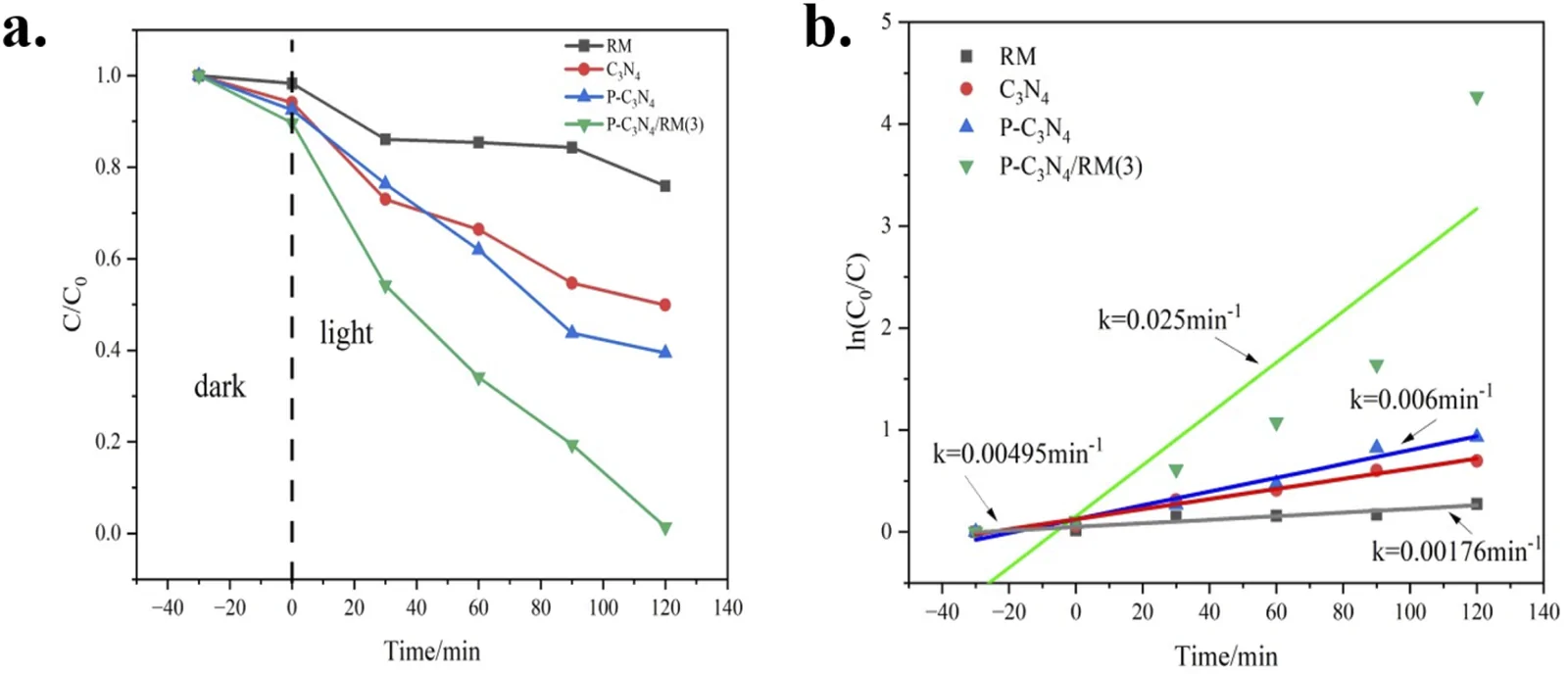
(a) Degradation efficiencies and (b) pseudo-first-order kinetic constants of pristine RM, g-C_3_N_4_, P-C_3_N_4_, and P-C_3_N_4_/RM (3) under visible-light irradiation in the presence of H_2_O_2_.

**Fig. 9:**
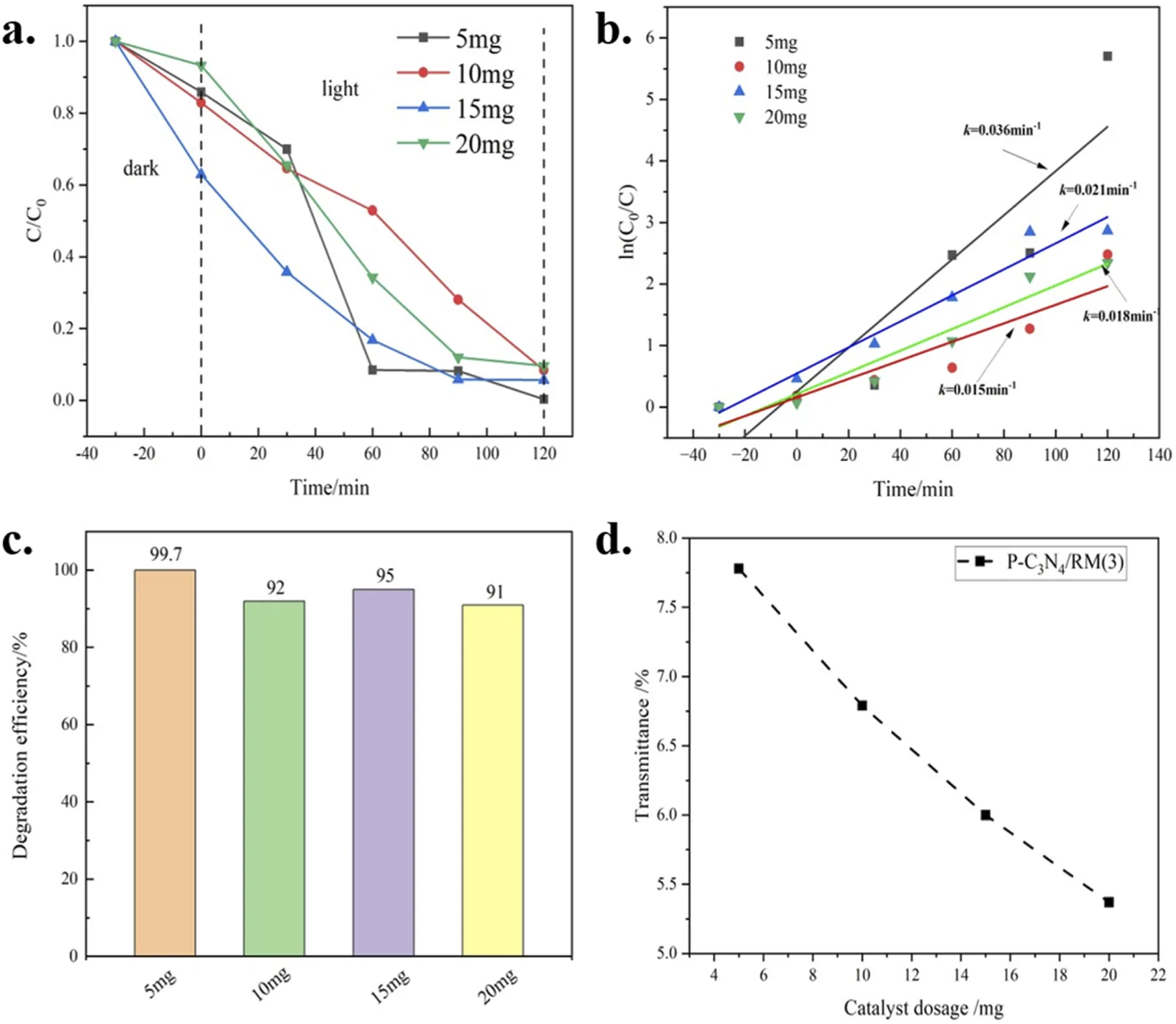
Effect of catalyst dosage on RhB photo-Fenton degradation: (a) degradation curves, (b) pseudo-first-order kinetic plots, (c) degradation efficiency, and (d) solution transmittance.

**Fig. 10:**
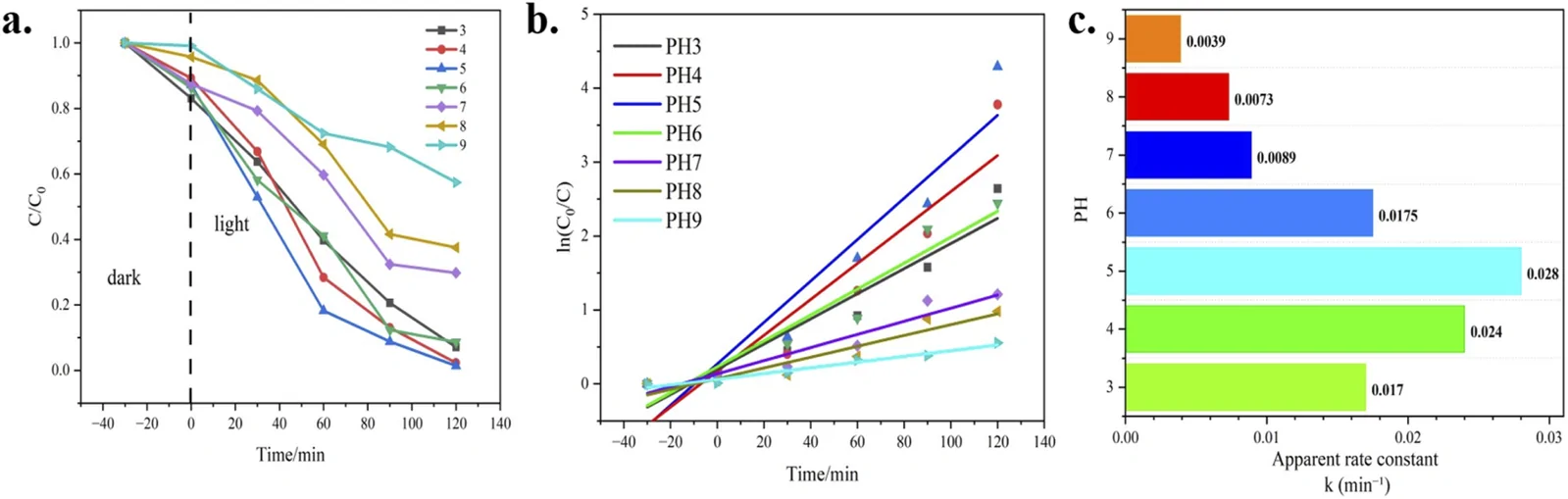
Effect of initial pH on RhB degradation over P-C_3_N_4_/RM (3) under visible-light/H_2_O_2_ conditions: (a) degradation curves at pH 3–9; (b) corresponding pseudo-first-order kinetic plots; and (c) apparent rate constants *k* at different pH values.

**Fig. 11:**
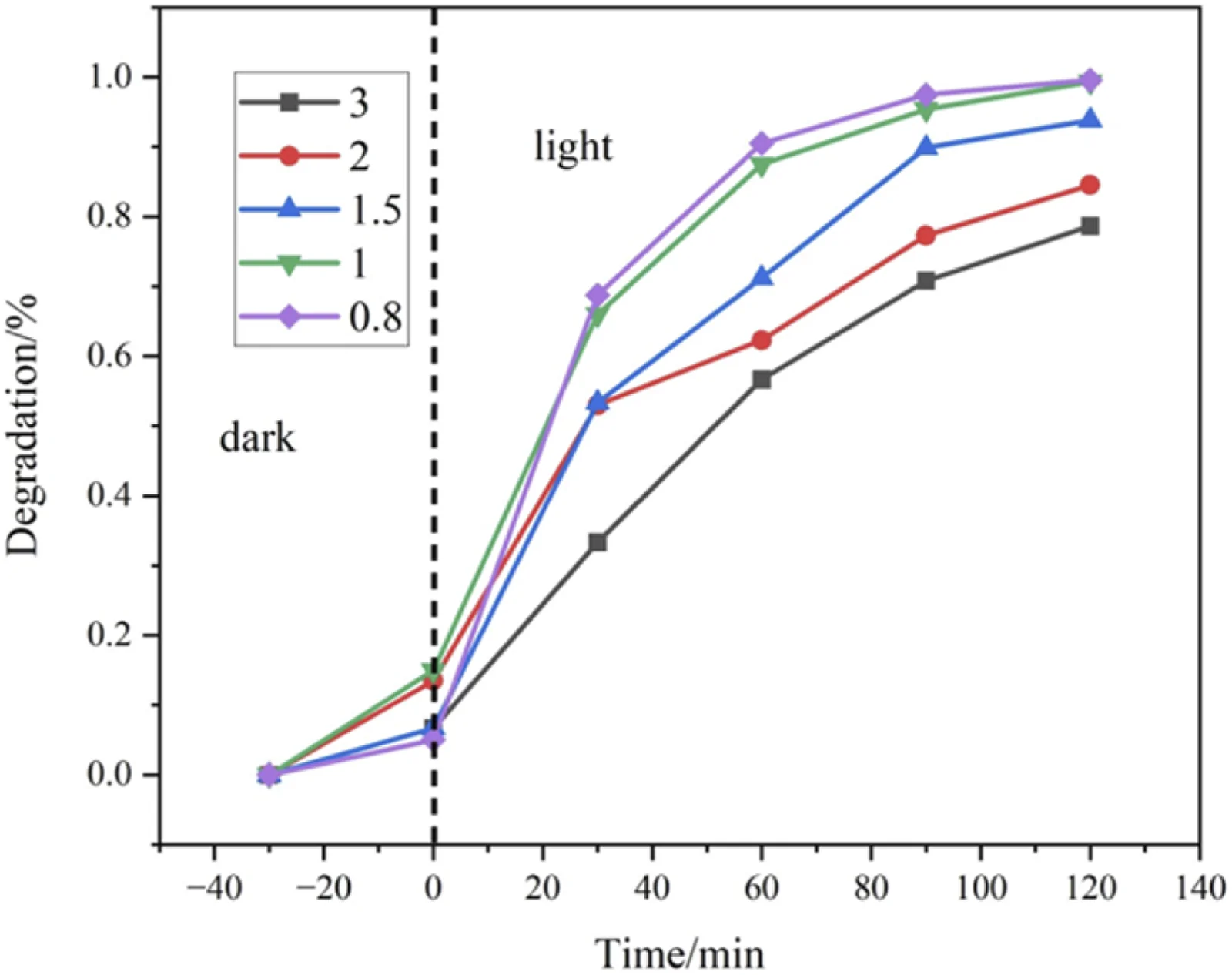
Effect of initial RhB concentration on photo-Fenton degradation over P-C_3_N_4_/RM (3).

#### 3.4.1. Comparison of photocatalytic, Fenton, and photo-Fenton systems

The degradation performance was first compared under photocatalytic, Fenton, and photo-Fenton conditions (Fig. 7a–c). The most pronounced decrease in *C*/*C*_0_ was observed for the photo-Fenton system, demonstrating a clear synergistic effect between visible-light photocatalysis and H_2_O_2_ activation. Among the tested materials, P-C_3_N_4_/RM exhibited the highest RhB removal efficiency in all three systems, and its advantage was particularly evident under photo-Fenton conditions. P-C_3_N_4_/RM (3) achieved nearly complete RhB removal, substantially outperforming P-C_3_N_4_ and pristine g-C_3_N_4_.

Pseudo-first-order kinetic analysis (Fig. 7d) further confirms the superior reaction kinetics of the P-C_3_N_4_/RM composites. P-C_3_N_4_/RM (3) exhibits the largest apparent rate constant among the tested samples and was therefore selected as the optimal catalyst for subsequent experiments.

To verify the synergistic effect between P-C_3_N_4_ and RM, the catalytic performances of pristine RM, g-C_3_N_4_, P-C_3_N_4_, and P-C_3_N_4_/RM (3) were evaluated under identical visible-light/H_2_O_2_ conditions (Fig. 8). As shown, the degradation efficiencies followed the order of P-C_3_N_4_/RM (3) > P-C_3_N_4_ > g-C_3_N_4_ > RM, with the corresponding apparent rate constants being 0.025, 0.006, 0.00495, and 0.00176 min^−1^, respectively. Notably, P-C_3_N_4_/RM (3) exhibited significantly higher activity than the individual components, demonstrating that the combination of P doping and RM coupling can effectively promote the catalytic performance through a pronounced synergistic effect.

The effect of catalyst dosage was subsequently investigated using P-C_3_N_4_/RM (3) (Fig. 9). A dosage of 5 mg gave the highest RhB degradation efficiency (99.7%). Increasing the catalyst mass to 10, 15, and 20 mg resulted in efficiencies of 92%, 95%, and 91%, respectively. Although a moderate increase in catalyst loading can provide more active sites, excessive suspended solids increase light scattering and absorption and thereby reduce light penetration through the reaction medium. The transmittance measurements in Fig. 9 directly support this interpretation: the solution transmittance decreased markedly as the catalyst dosage increased from 5 to 20 mg. Thus, the benefit of additional active sites at high catalyst loadings is outweighed by the detrimental light-screening effect, leading to lower generation rates of photocarriers and reactive oxygen species. A dosage of 5 mg therefore provides the best compromise between accessible active sites and efficient photon utilization under the present conditions.

#### 3.4.2. Effect of initial pH

The influence of initial pH on RhB degradation was evaluated in the pH range of 3–9 (Fig. 10). As shown, both the degradation efficiency and the apparent rate constant were maximized at pH 5 and remained at relatively high levels between pH 3 and 6. In contrast, a gradual decline in catalytic activity was observed as the pH increased from 7 to 9. The corresponding apparent rate constants at pH 3, 4, 5, 6, 7, 8, and 9 were 0.017, 0.024, 0.028, 0.0175, 0.0089, 0.0073, and 0.0039 min^−1^, respectively. The deterioration in degradation performance under alkaline conditions is mainly ascribed to the lower stability of H_2_O_2_ and the precipitation of Fe^3+^ into hydroxide species, which impede the Fe^3+^/Fe^2+^ redox cycle and thus decrease ·OH production. Overall, the catalyst maintains high activity in the pH 3–6 range and remains active in the pH 7–9 range.

#### 3.4.3. Effect of initial RhB concentration

The initial RhB concentration strongly influenced the photo-Fenton performance (Fig. 11). As the initial dye concentration increased, both the degradation efficiency and the apparent reaction rate decreased. At the lower tested concentrations, RhB was almost completely removed within 120 min, whereas at the highest tested concentration the degradation efficiency after 120 min was only 78%. This decrease can be attributed to stronger inner-filter/light-screening effects, competitive occupation of surface active sites, and increased consumption of reactive oxygen species at higher pollutant loadings. The catalyst therefore shows particularly strong potential for the treatment of low-concentration dye wastewater.

To place the performance of the present catalyst in context, P-C_3_N_4_/RM was compared with previously reported g-C_3_N_4_-based systems for RhB degradation (Table 2). The present material operates at a comparatively low catalyst dosage while maintaining a competitive apparent reaction rate constant, indicating efficient utilization of the catalyst. The dosage-dependent transmittance results further emphasize the importance of balancing active-site density against light attenuation in slurry photo-Fenton reactors.

**Table 2:** Comparison of representative g-C_3_N_4_-based catalysts for RhB degradation

Catalyst	Reaction system	Catalyst dosage	Apparent rate constant *k* (min^−1^)	Reference
Benzene/K co-doped g-C_3_N_4_	Photo-Fenton	0.5 g L^−1^	0.0446	8
α-FeOOH/CDs/p-GCN	Photo-Fenton	0.2 g L^−1^	0.021	9
P-doped g-C_3_N_4_	Photocatalysis	0.5 g L^−1^	∼0.30	10
**P-C**_**3**_ **N**_**4**_ **/RM**	**Photo-Fenton**	**0.1 g L** ^**−1**^	**0.036**	**This work**

#### 3.4.4. Effect of coexisting inorganic species and humic acid

The resistance of P-C_3_N_4_/RM (3) to common water-matrix constituents was evaluated by introducing different inorganic salts and humic acid (HA) (Fig. 12). The addition of NaCl, NaHCO_3_, NaNO_3_, or HA caused only moderate inhibition of RhB degradation. By contrast, NaH_2_PO_4_·2H_2_O produced a pronounced decrease in catalytic activity. This behavior may result from competitive coordination of H_2_PO_4_^−^ with iron-based active sites, which interferes with Fe^2+^/Fe^3+^ cycling and H_2_O_2_ activation. Phosphate species can also scavenge highly reactive ·OH, thereby decreasing the effective oxidant flux. Overall, the catalyst retains substantial activity in the presence of several representative inorganic anions and natural organic matter, indicating a useful degree of tolerance toward complex water matrices.

**Fig. 12:**
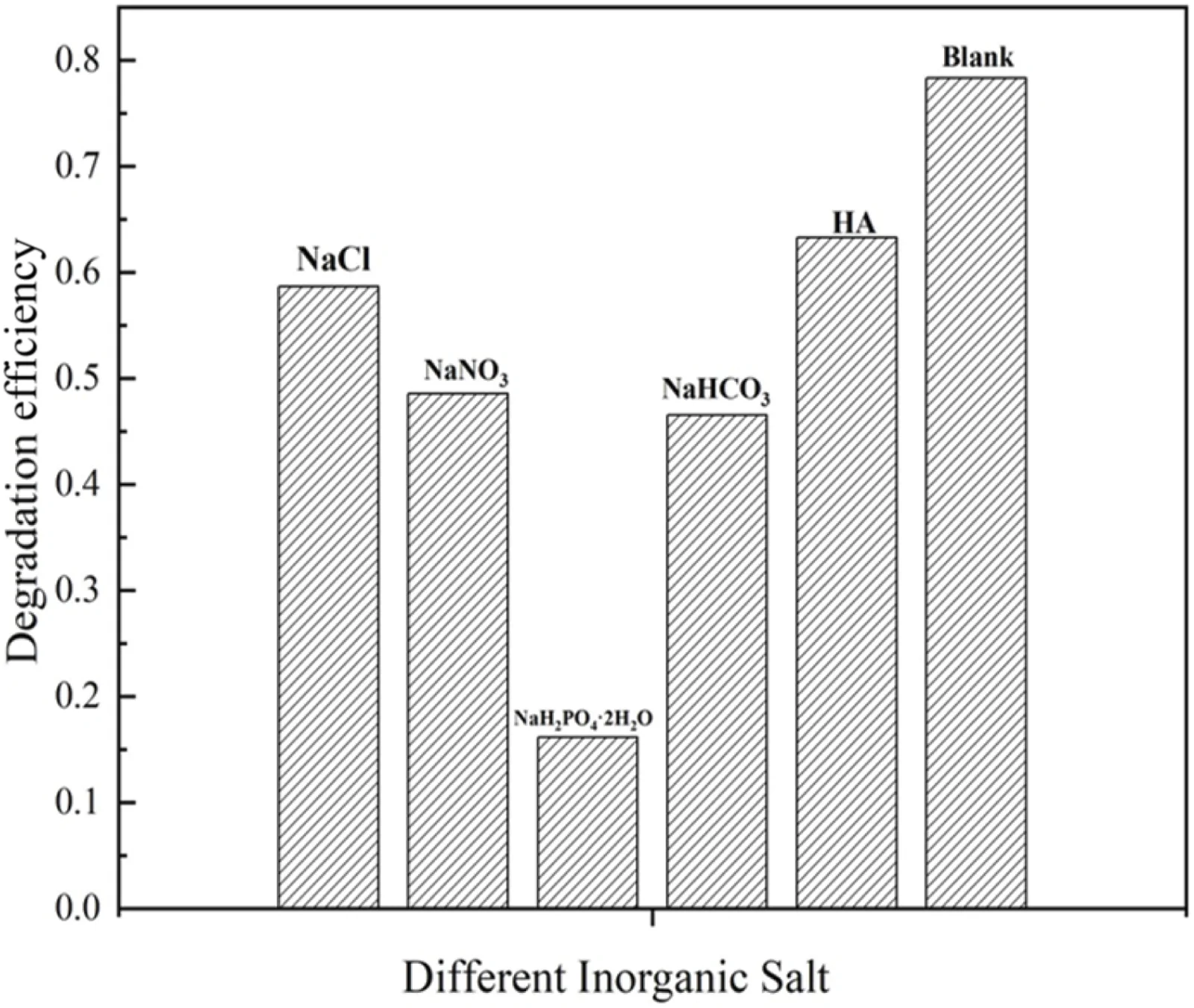
Effect of coexisting inorganic salts and humic acid on RhB degradation over P-C_3_N_4_/RM (3).

### 3.5. Reusability and structural stability

The reusability of P-C_3_N_4_/RM (3) was assessed over three consecutive RhB degradation cycles (Fig. 13a). After each cycle, the reaction suspension was centrifuged at 8000 rpm, and the recovered catalyst was washed three times with anhydrous ethanol and deionized water, followed by drying at 60 °C for 12 h prior to reuse. P-C_3_N_4_/RM (3) maintained a high RhB removal efficiency during repeated use, achieving 97% in the first cycle, 97% in the second cycle, and 96% in the third cycle.

**Fig. 13:**
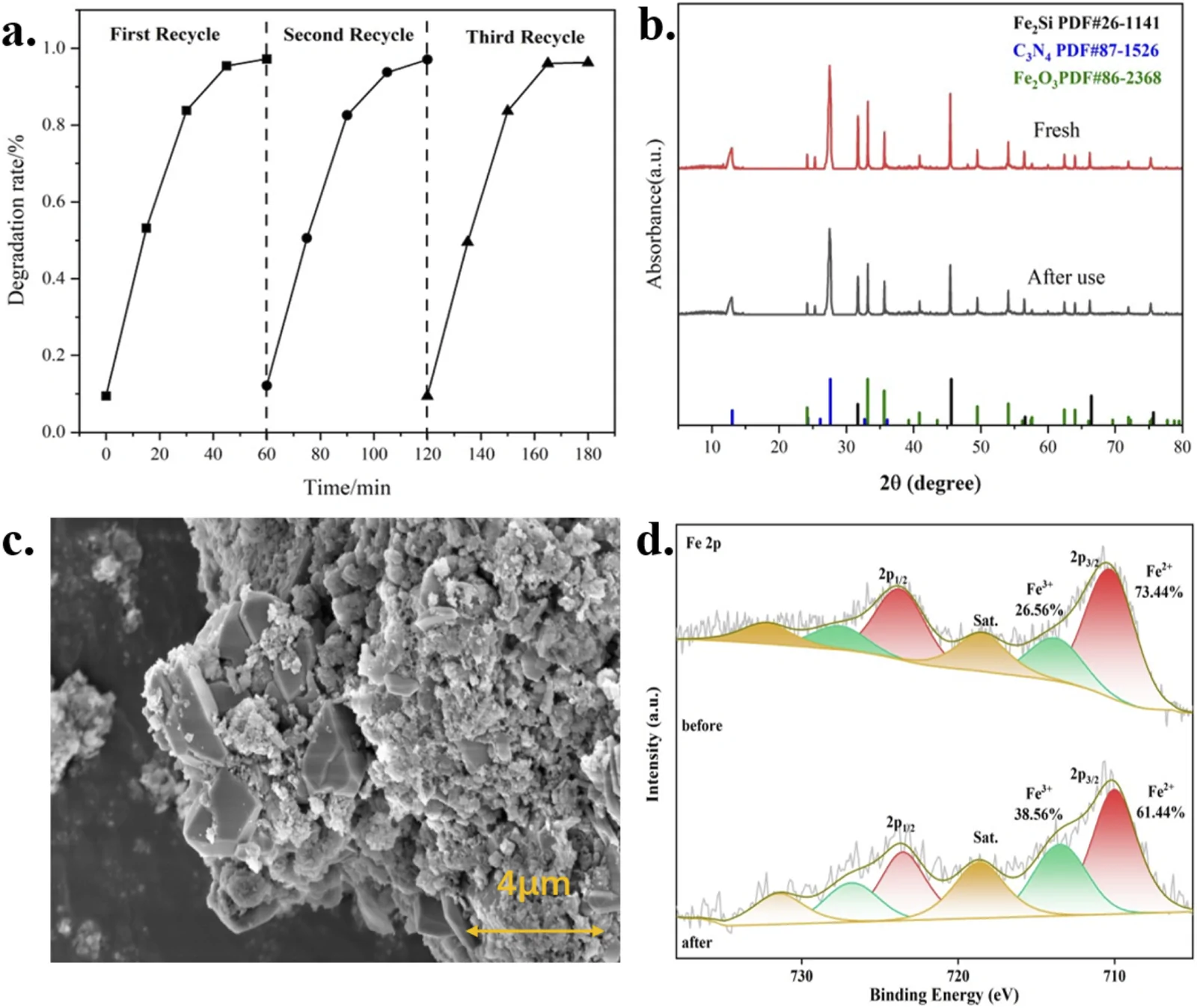
Reusability and structural stability of P-C_3_N_4_/RM (3): (a) cyclic RhB degradation profiles, (b) XRD patterns before and after use, (c) SEM image after cycling, and (d) Fe 2p XPS spectra before and after cycling.

The SEM image of the used catalyst (Fig. 13c) reveals that the porous morphology is largely preserved after cycling, with no obvious structural collapse or significant grain growth.

To further evaluate the structural stability of the composite, the XRD patterns before and after cycling were compared (Fig. 13b). The crystallite sizes of the g-C_3_N_4_ and Fe_2_O_3_ phases were estimated using the Scherrer equation, *D* = *Kλ*/(*β*  cos *θ*). The g-C_3_N_4_ (100) reflection at about 2*θ* = 12.96° gave a crystallite size of about 197.7 nm both before and after the reaction. The Fe_2_O_3_ (110) reflection at about 2*θ* = 35.66° gave about 97.1 nm before and about 97.1 nm after the reaction. The variation in crystallite size is within the experimental error, confirming that the composite preserves its crystal structure during repeated cycling.

To further examine the Fe^3+^/Fe^2+^ cycling during the photo-Fenton reaction, the Fe 2p XPS spectra before and after cycling were compared (Fig. 13d). Before the reaction, the fractions of Fe^2+^ and Fe^3+^ were 73.44% and 26.56%; after cycling, they changed to 61.44% and 38.56%. The decrease in Fe^2+^ and increase in Fe^3+^ indicate that Fe^2+^ participated in H_2_O_2_ activation and was oxidized to Fe^3+^ during the reaction. Combined with ESR and radical trapping results, these data confirm that Fe^2+^ participates in H_2_O_2_ activation and is regenerated through the reduction of Fe^3+^ by photogenerated electrons, sustaining the Fe^3+^/Fe^2+^ cycle.

Overall, these results demonstrate that P-C_3_N_4_/RM (3) possesses excellent structural robustness and recyclability, highlighting its potential for practical application in pollutant degradation.

### 3.6. Identification of reactive species

Reactive-species trapping experiments were performed using triethanolamine (TEOA) as a hole (h^+^) scavenger, isopropanol (IPA) as a hydroxyl-radical (·OH) scavenger, and l-histidine as a singlet-oxygen (^1^O_2_) quencher. In addition, N_2_ purging was used to remove dissolved oxygen and suppress the formation of superoxide species (·O_2_^−^). The addition of IPA or l-histidine strongly inhibited RhB degradation, whereas TEOA addition or N_2_ purging caused only a limited decrease. These results indicate that ·OH and ^1^O_2_ are the dominant reactive species, while h^+^ and ·O_2_^−^ make smaller direct contributions. Electron spin resonance (ESR) measurements provided further evidence: no characteristic radical signals were detected in the dark, whereas under light irradiation in the presence of H_2_O_2_, the characteristic TEMP-^1^O_2_ triplet and the DMPO-·OH 1 : 2 : 2 : 1 quartet were observed. Thus, visible-light irradiation promotes H_2_O_2_ activation and the concurrent generation of ·OH and ^1^O_2._

The high activity of P-C_3_N_4_/RM (3) can therefore be attributed not only to improved charge separation but also to its ability to activate H_2_O_2_ and promote multiple oxidative pathways dominated by ·OH and ^1^O_2_. This coupled mechanism helps sustain degradation performance in chemically complex aqueous environments (Fig. 14).

**Fig. 14:**
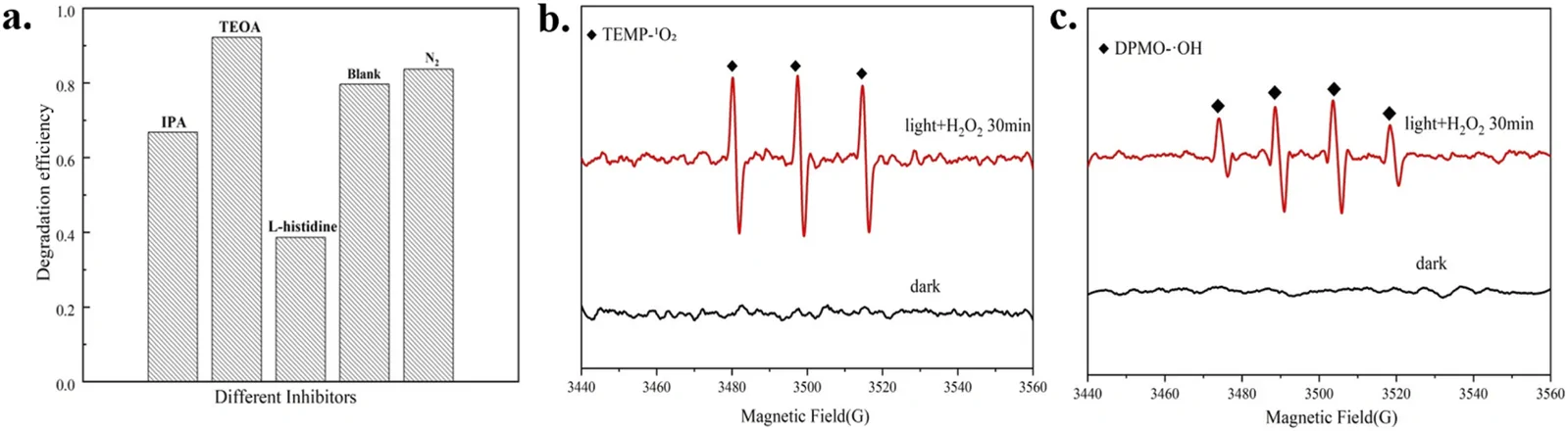
Reactive-species identification for P-C_3_N_4_/RM (3): scavenger experiments and ESR spectra of ·OH and ^1^O_2_.

### 3.7. Proposed photo-Fenton degradation mechanism

Changes in the UV-vis absorption spectra during RhB degradation were used to examine the transformation pathway (Fig. 15a). RhB degradation involves two principal processes: *N*-deethylation and destruction of the conjugated chromophore. The main visible absorption band at 554 nm arises from the conjugated xanthene structure, while an additional UV band is associated with the aromatic framework. As the reaction proceeds, the 554 nm band rapidly decreases and the solution gradually becomes colorless, indicating destruction of the chromophore and formation of intermediate products through *N*-deethylation. New transient absorption features around 300 and 352 nm are consistent with the formation and subsequent oxidation of smaller organic intermediates. The final products are inferred to include low-molecular-weight organic acids that are ultimately mineralized to H_2_O, CO_2_, and inorganic ions. The mineralization results are consistent with this pathway, with approximately 95% total organic carbon (TOC) removal and 93% chemical oxygen demand (COD) removal.

**Fig. 15:**
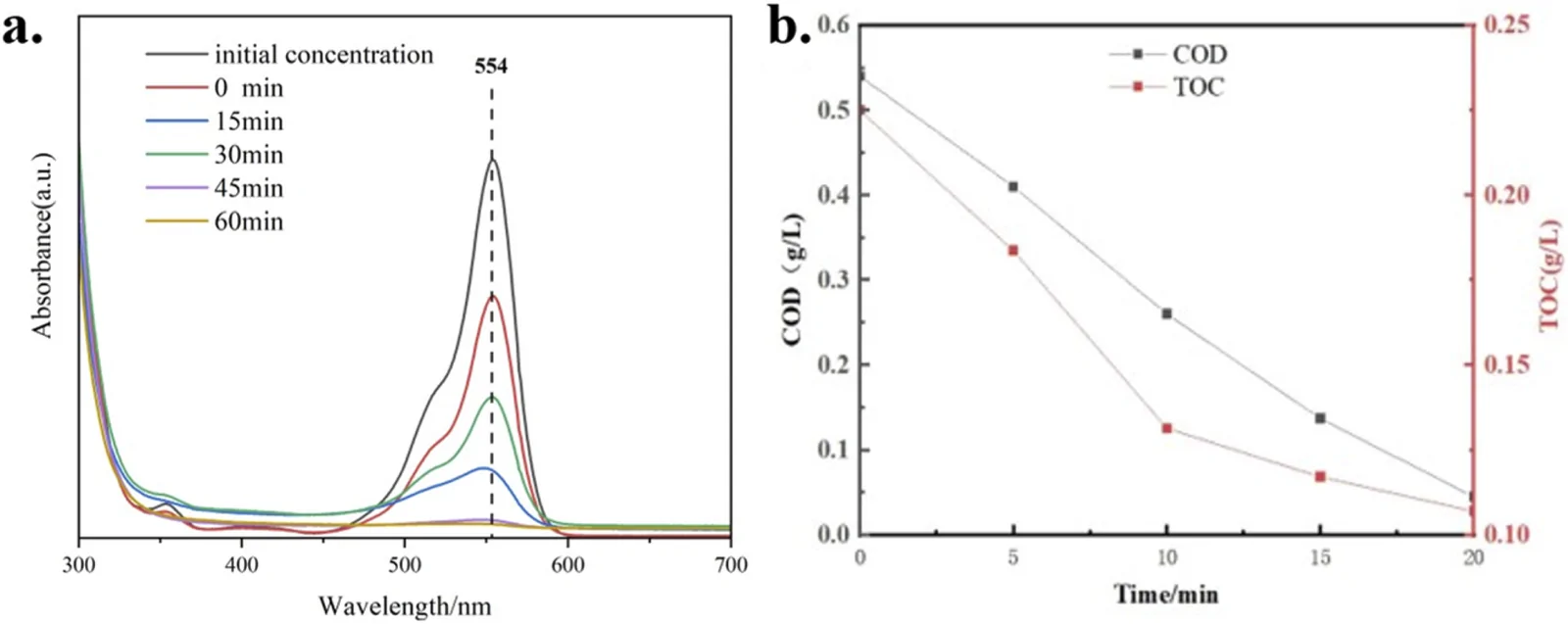
Evolution of RhB during degradation over P-C_3_N_4_/RM: (a) time-dependent UV-vis spectra and (b) TOC and COD removal from the residual solution.

Based on the structural, optical, electrochemical, trapping, and ESR results, a plausible photo-Fenton mechanism is proposed (Fig. 16). Phosphorus incorporation into the g-C_3_N_4_ framework introduces local electronic states, narrows the band gap, extends visible-light absorption, and promotes carrier delocalization and transport. The conduction-band potential of P-C_3_N_4_ is shifted to a more negative value (approximately −0.33 V *vs.* NHE), increasing the reducing ability of photogenerated electrons. Electrons transferred to the RM component can participate in two coupled pathways. First, they promote Fe^3+^/Fe^2+^ cycling by reducing surface Fe^3+^ to Fe^2+^; Fe^2+^ then reacts with H_2_O_2_ to generate strongly oxidizing ·OH and is reoxidized to Fe^3+^, thereby closing the Fenton cycle. Consumption of transferred electrons in this process also favors continued interfacial charge separation. Second, transferred electrons can reduce dissolved O_2_ to ·O_2_^−^, which can subsequently participate in pathways leading to ^1^O_2_ formation. Meanwhile, holes remaining in the valence band of P-C_3_N_4_ may directly oxidize organic molecules or contribute to ·OH generation. The combined action of ·OH, ^1^O_2_, and, to a lesser extent, h^+^ progressively breaks down the RhB molecular framework into smaller intermediates and ultimately mineralized products. The overall reaction pathway can be summarized as follows:P-C_3_N_4_ + *hν* → P-C_3_N_4_ (h^+^ + e^−^)P-C_3_N_4_ (e^−^) + RM → P-C_3_N_4_ + RM (e^−^)RM (e^−^) + O_2_ → ·O_2_^−^·O_2_^−^ + h^+^ → ^1^O^2^Dye + ·OH/^1^O_2_/h^+^ → small molecules or inorganic ions

**Fig. 16:**
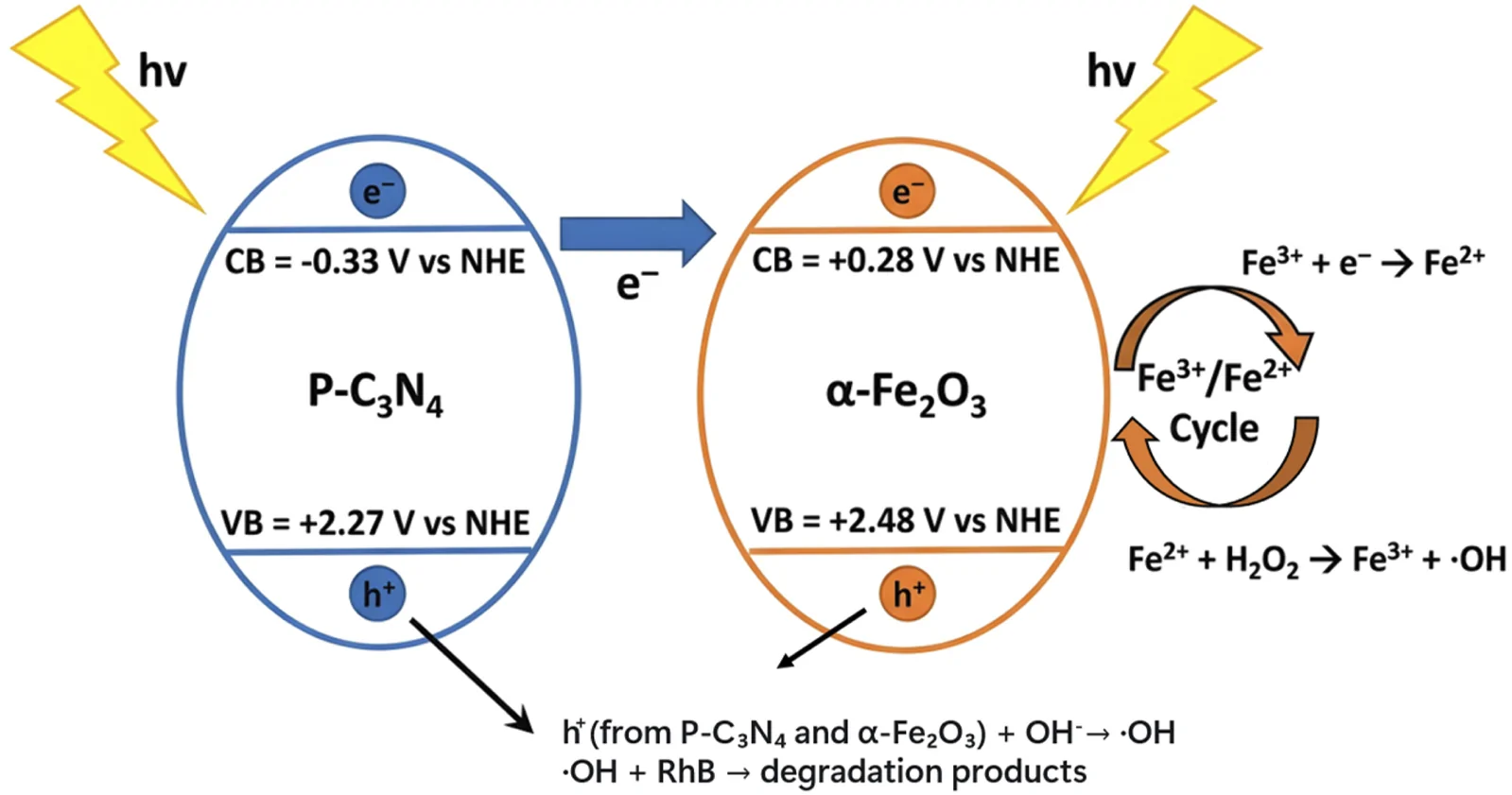
Proposed mechanism for RhB degradation in the P-C_3_N_4_/RM photo-Fenton system.

## 4. Conclusions

A P-C_3_N_4_/RM heterogeneous photo-Fenton catalyst was successfully prepared using an impregnation-assisted thermal route. Structural characterization confirmed phosphorus incorporation into the carbon nitride framework and intimate contact between RM-derived particles and the lamellar P-C_3_N_4_ matrix, resulting in effective modulation of the electronic structure and photoelectrochemical behavior.

Among the prepared materials, P-C_3_N_4_/RM (3), corresponding to an RM addition of 0.8 g during synthesis, exhibited the best performance. In the visible-light/H_2_O_2_ system, it achieved 99.7% RhB degradation within 120 min of irradiation, with an apparent rate constant of 0.036 min^−1^. The system maintained high efficiency in the pH 3–6 range and remained active in the pH 7–9 range. Radical-quenching and ESR analyses identified ·OH and ^1^O_2_ as the major reactive species responsible for oxidation, and Fe 2p XPS before and after cycling confirmed the Fe^3+^/Fe^2+^ cycling during the reaction.

The catalyst showed good tolerance to variations in solution chemistry and maintained high activity in the presence of several common inorganic species and humic acid. A catalyst dosage of 5 mg was optimal under the tested conditions, while excessive loading induced a light-screening penalty. After three consecutive cycles, the RhB degradation efficiency remained 96%, demonstrating good reusability. The integration of phosphorus-modified carbon nitride with iron-rich red mud therefore provides a promising route for converting an abundant industrial residue into a functional heterogeneous photo-Fenton catalyst for dye-wastewater treatment.

## Conflicts of interest

There are no conflicts to declare.

## Data Availability

All data supporting this article are included in the main manuscript.
